# Consistency assessment of milk fat and protein percentages across 3 daily milkings in Holstein and Jersey dairy herds

**DOI:** 10.3168/jdsc.2025-0748

**Published:** 2025-06-03

**Authors:** Xiao-Lin Wu, Malia J. Caputo, Asha M. Miles, Ransom L. Baldwin, Steven Sievert, Jay Mattison, John B. Cole, Javier Burchard, João Dürr

**Affiliations:** 1 Council on Dairy Cattle Breeding, Bowie, MD 20716; 2 Department of Animal and Dairy Sciences, University of Wisconsin, Madison, WI 53511; 3 USDA Animal Genomics and Improvement Laboratory, Beltsville, MD 20705; 4 National Dairy Herd Information Association, Verona, WI 53593; 5 Department of Animal Science, North Carolina State University, Raleigh, NC 27607; 6 Department of Animal Sciences, Donald Henry Barron Reproductive and Perinatal Biology Research Program, and the Genetics Institute, University of Florida, Gainesville, FL 32610

## Abstract

Dairy cattle milking test plans in the United States and globally have evolved substantially since the 1960s toward cost-effective sampling methods. Test-day recording frequencies vary, adapting to the specific management needs of different herds. Typically, a cow is milked twice or more daily; however, milk fat and protein percentages are commonly assessed from single-milking samples. In this paper, we introduced intraclass correlation coefficients to determine the consistency of intraday milk fat and protein percentages across multiple milkings within the same cow. This metric extends beyond simple pairwise correlations, enabling robust comparisons across multiple milkings. Various forms of intraclass correlations are also demonstrated. Our results show that although protein percentages exhibit high consistency, fat percentages display notable variability throughout the test day. Hence, adjustment factors for milk fat percentage should differ according to individual milkings and consider the effects of the milking interval, DIM, and parity. Overall, the results demonstrate the utility of intraclass correlation as a consistency measure, providing a valuable tool for assessing the data quality of milk components for dairy breeding and management decisions.

## Highlights

• Intraclass correlation enables robust comparisons across multiple milkings.
• Milk protein percentages exhibited high consistency across milkings.
• Milk fat percentages varied notably, needing adjustments or rotative sampling.
• Intraclass correlation proved to be a valuable tool for assessing milking data quality.

## Graphical Abstract

**Figure:**
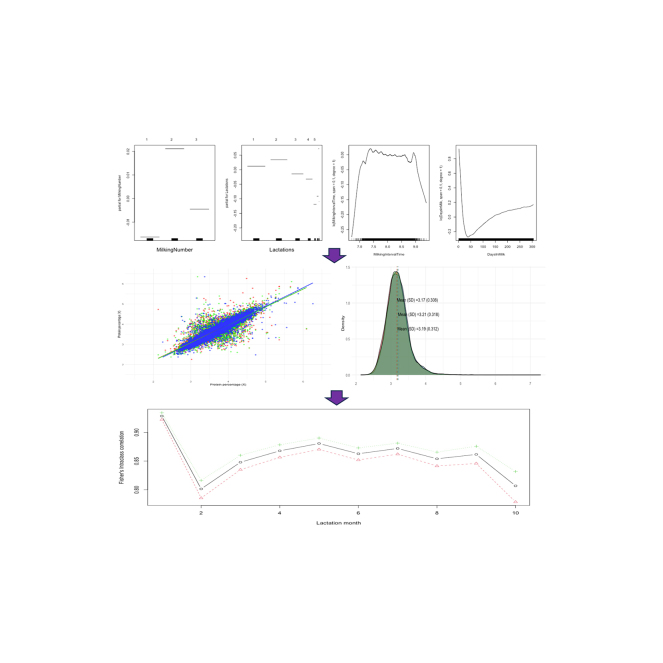
**Summary:** Dairy cattle milking test plans in the United States and globally have evolved significantly toward cost-effective sampling methods since the 1960s. Test-day recording frequencies vary, adapting to the specific management needs of different herds. Typically, a cow is milked 2 or more times daily. Still, milk fat and protein percentages are commonly assessed from 1 milking sample, assuming the percentages are stable throughout the day. However, milk compositional percentages vary across milkings, and a reliability measure is needed to assess milk component data quality. We used intraclass correlation coefficients to assess consistency across multiple milkings within a cow and applied them to explore data quality of milking components in 4 dairy farms.

Milking test plans have substantially transformed toward cost-efficient milk sampling strategies since the 1960s in the United States and other countries, mainly to minimize costs associated with DHIA supervisor visits (Wu et al., 2023a). The frequency of test-day recordings varies, adapting to various herd management strategies. Typically, a cow is milked twice or more daily, yet not all milkings are weighed and sampled. Fat and protein percentages are often assessed on single-milking samples, and adjustments are made assuming stable milk compositions across multiple milkings daily.

Two metrics are relevant to guide data quality control: reliability and accuracy. The former assesses the consistency of multiple measurements, indicating whether results are reproducible under the same conditions. The latter reflects how closely fat and protein percentages from a single milking align with those derived from the whole daily milk yields. Statistically, precision is synonymous with reliability, measuring random errors, whereas accuracy is interchangeably used with validity, measuring systematic error or the closeness of measurements to the “true” values. In this study, we propose using the intraclass correlation coefficient (**ICC**; Bartko, 1966) as a consistency measure for single-milking fat and protein percentages and apply it to accessing the data quality of milk components in 4 selected dairy farms.

Intraclass correlation is not new, because human and animal geneticists have used ICC for decades in genetics studies. The degree of resemblance between family relatives enables the estimation of additive genetic variance, with the proportionate of additive variance (heritability) serving as a primary determinant of optimal breeding methods for genetic improvement (Falconer and Mackay, 1996). By employing ANOVA, the total observed variance can be partitioned into between-family and within-family variances. The between-family component reflects the variance of the groups' “true” means relative to the population mean, whereas the within-family component captures the variance of individuals around their family's true mean. Consequently, the degree of resemblance can be expressed by the between-family component as a proportion of the total variance, corresponding to the ICC for families. Interclass correlation also approximates repeatability without distinguishing additive genetic effects from permanent environmental impact, assuming fat (protein) percentage is the same trait across multiple milkings (Falconer and Mackay, 1996).

Fisher (1954) first introduced the ICC as a modification of the Pearson correlation coefficient. To apply it in evaluating the consistency of multiple measurements from different milkings, consider *n* cows, each assessed for fat (or protein) percentage during 3 milkings daily on a test day. The intraclass correlation is defined as

[1] $$\begin{array}{l} r=\frac{1}{3ns^{2}}\sum_{i=1}^{n}\;\{\left(x_{i1}-\bar{x}\right)\left(x_{i2}-\bar{x}\right)+\left(x_{i1}-\bar{x}\right)\left(x_{i3}-\bar{x}\right)\;\\ +\;\left(x_{i2}-\bar{x}\right)\left(x_{i3}-\bar{x}\right)\}, \end{array}$$

where $\bar{x}=\frac{1}{3n}\sum_{i=1}^{n}\;\left(x_{i1}+x_{i2}+x_{i3}\right)$ and $s^{2}=\frac{1}{3n}\sum_{i=1}^{n}\;\left\{{\left(x_{i1}-\bar{x}\right)}^{2}+{\left(x_{i2}-\bar{x}\right)}^{2}+{\left(x_{i3}-\bar{x}\right)}^{2}\right\},$ for *i* = 1, ..., *n* individuals. The correlation ranges from 0 to 1, with a high value indicating strong consistency and a low value suggesting significant variation across multiple milkings. Fisher (1954) also proposed assessing the sampling errors of ICC by employing a logarithm transformation of a rational function of ICC for cases with *k* > 2 groups. In this study, we estimated ICC sampling errors using bootstrapping.

The number of cross-products in this expression grows as the number of milkings (*k*) increases, leading to substantially increased computation with Equation 1. Attributed to Harris (1913), an alternative equivalent form of ICC, yet simpler, is the following:

[2] $$r=\frac{k}{k-1}\times \frac{\sum_{i=1}^{n}\;{\left({\bar{x}}_{i}-\bar{x}\right)}^{2}}{ns^{2}}-\frac{1}{k-1},$$

where ${\bar{x}}_{i}=\frac{1}{k}\sum_{j=1}^{k}\;x_{ij},$ for *j* = 1, ..., *k* milkings daily. For a large *k*, this ICC is approximately equal to

[3] $$r\approx \frac{\sum_{i=1}^{n}\;{\left({\bar{x}}_{i}-\bar{x}\right)}^{2}}{ns^{2}}.$$

Modern ICC is calculated by mean squares based on ANOVA (Shrout and Fleiss, 1979). Equation 3 can also be interpreted as the fraction of the total variance due to variation between groups. Modeling strategies vary; a one-way ANOVA model assumes random effects only for the subjects (i.e., cows), whereas a 2-way ANOVA model can account for both subjects and raters (i.e., milkings) as sources of variability. Consider a one-way ANOVA model. Suppose we have a set of measurements *y_ij_*, where *i* = 1, 2, …, *n* is the number of cows, and *j* = 1, 2, …, *k* is the number of repeated measurements daily for each cow. Assume equal true values across multiple measurements per cow (*a_i_*). The observed measurement *y_ij_* can be modeled as

[4] *y_ij_* = *μ* + *a_i_* + *w_ij_*.

Here, *a_i_* is defined as the difference from the overall mean (*μ*) of the true value associated with the *i*th animal, assumed to be normally distributed with a zero mean and variance $\sigma_{a}^{2},$ and *w_ij_* is a residual term, also assumed to be normal with a zero mean and variance $\sigma_{w}^{2}.$*w*. The ICC under this scenario is computed as follows:

[5] $$ICC1=\frac{\sigma_{a}^{2}}{\sigma_{a}^{2}+\sigma_{w}^{2}}=\frac{BMS-WMS}{BMS+\left(k-1\right)WMS}.$$

The one-way ANOVA model estimates between-subject mean square (**BMS**) and within-subject mean square (**WMS**), which are then used to $\sigma_{a}^{2}$ and $\sigma_{w}^{2}.$ When $\sigma_{w}^{2}$ is small, ICC1 approaches 1, indicating high measurement consistency. When $\sigma_{w}^{2}$ is large, ICC1 approaches 0, suggesting low consistency among single intraday measurements per cow.

The 2-way ANOVA model separates the effects in *w_ij_* due to multiple measurements *b_j_*, the interaction between measurements and animals (*ab*)*_ij_*, and random errors e*_ij_*, respectively.

[6] *y_ij_* = *μ* + *a_i_* + *b_j_* + (*ab*)*_ij_* + e*_ij_*.

This analysis partitions the within-animal sum of squares into a between-measurement sum of squares and an error sum of squares. Thus, it additionally gives between-measurement mean square (**JMS**) and random error mean square (**EMS**), compared with Equation 4.

Various types of ANOVA-based ICC have been defined (Shrout and Fleiss, 1979). Briefly, ICC1 measures absolute agreement for fat or protein percentage across single milkings based on a one-way random effects model; ICC2 assesses consistency among milkings when both cows and milkings are considered random effects, based on a 2-way random effects model; ICC3 evaluates consistency for single milkings while treating milkings are fixed effects, employing a 2-way mixed-effects model. For the latter 2 scenarios, ICC are computed as follows:

[7] ICC2=σa2σa2+σb2+σI2+σe2=BMS−EMSBMS+(k−1)EMS+k(JMS−EMS)/BMS+(k−1)EMS+k(JMS−EMS)nn,

[8] ICC3=σa2−σI2/σa2−σI2(k−1)(k−1)σa2+σI2+σe2=BMS−EMSBMS+(k−1)EMS.

Here, ICC2 differs from ICC3 regarding the assumption about *b_j_* and (*ab*)*_ij_* in Equation 6. With ICC2, *b_j_* is assumed to be a random variable following a normal distribution with a zero mean and variance $\sigma_{b}^{2},$ whereas with ICC3, *b_j_* is a fixed effect subject to the constraint: $\sum_{j=1}^{k}\;b_{j}=0.$ This implies that $\sigma_{b}^{2}=\frac{1}{k-1}\sum_{j=1}^{k}\;b_{j}^{2}.$ With ICC2, all the components can be assumed to be mutually independent, each with a zero mean and variance $\sigma_{I}^{2},$ for *i* = 1, …, *n* and *j* = 1, …, *k*. In contrast, the ICC3 model assumes independence only for interaction components involving different animals; within the same animal, interactions must satisfy the constraint $\sum_{j=1}^{k}\;{\left(ab\right)}_{ij}=0.$ For simplicity, assume that measurement errors of fat and protein percentages arise solely from the laboratory analyses. The difference between ICC2 and ICC3 can be viewed in such a way that the ICC3 model assumes analyzers (machines and technicians) are fixed, whereas the ICC2 model allows analyzers to vary randomly. With ICC1, the analyzers are also considered random, selected from a larger available set (*m* ≥ *k*).

Further, consistency can be evaluated for the mean across multiple measurements. For instance, ICC1k measures absolute agreement for the average of *k* milkings using a one-way random effects model, assuming milkings are random effects. Typically, averaging across multiple milkings enhances consistency. The ICC2k metric evaluates consistency for the average of *k* milkings, assuming both cows and milkings are random effects within a 2-way random effects model. The ICC3k metric assesses consistency for the average of *k* milkings while treating milkings as fixed effects, using a 2-way mixed-effects model. Their ANOVA-based formulas are as follows:

[9] $$ICC1k=\frac{BMS-WMS}{BMS},$$

[10] ICC2k=BMS−EMSBMS+(JMS−EMS)/BMS+(JMS−EMS)nn,

[11] $$ICC3k=\frac{BMS-EMS}{BMS}.$$

We refer to Equation 1 as Fisher's ICC and the latter forms (Equations 5, 6, 7, 8, 9, 10, and 11) as ANOVA-based ICC.

We applied the ICC approach to assess the consistency of fat and protein percentages in 3-milking-daily samples from 4 dairy farms: farm 1 (Holstein) and farm 3 (Jersey) in State A, farm 2 (Jersey) in state B, and farm 4 (Holstein) in state C. These farms were numbered according to the order in which they participated in the present study from 2023 to 2024. Typically, the 3 milkings occurred in the early morning (0400–0600 h), midday (1200–1400 h), and late evening (2000–2200 h), with some variation in exact timing for practical convenience. Data cleaning removed redundant, missing, and incomplete data, retaining 48,921 milking records from farm 1, 39,132 records from farm 2, 36,783 records from farm 3, and 40,827 records from farm 4 for subsequent analyses.

Approximately 90% to 92% of the milking records from farms 1, 3, and 4 and ∼75% of the records from farm 2 were obtained from cows in lactations 1 to 4. The mean DIM, along with the 95% CI, were 125 (20–309) in farm 1, 84 (6–203) in farm 2, 156 (3–357) in farm 3, and 141 (9–300) in farm 4. The distributions of milking interval time were unimodal in the 2 Holstein herds (farms 1 and 4). In comparison, the 2 Jersey herds (farms 2 and 3) exhibited multimodal distributions, likely due to greater variability in milking interval durations (figures not presented). The mean (SD) of milking intervals (in hours) for the 3 milkings were 7.25 to 8.79 (0.43–0.50) in farm 1, 7.10 to 8.53 (0.32–1.64) in farm 2, 7.94 to 8.20 (0.62–0.97) in farm 3, and 7.82 to 8.05 (0.19–0.26) in farm 4.

On average, each milking contributed approximately one-third of the daily milk yield. The mean (SD) of proportional daily milk yields for the 3 milkings were 0.31 to 0.37 (0.03–0.04) in farm 1, 0.30 to 0.36 (0.04–0.07) in farm 2; 0.33 to 0.34 (0.05) in farm 3, and 0.32 to 0.34 (0.03–0.04) in farm 4. The reciprocals of these proportional daily yields provided empirical estimates of multiplicative correction factors (**MCF**) for adjusting daily milk yields (Wu et al., 2023b). The mean (SD) of empirical MCF across the 3 milkings were 2.75 to 3.30 (0.28–0.36) in farm 1, 2.48 to 2.81 (0.35–0.70) in farm 2, 2.96 to 3.11 (0.25–0.26) in farm 3, and 2.99 to 3.08 (0.36–0.45) in farm 4. The MCF fundamentally depend on milking interval. Assuming precisely equal intervals between the 3 milkings, the expected MCF would be 3.

Across the 4 farms, fat percentages were higher and exhibited substantially greater variation than protein percentages among the 3 milkings. The means (SD) of fat percentages for the 3 milkings were 3.87% to 4.31% (0.69%–0.71%) in farm 1, 4.36% to 5.09% (0.71%–0.78%) in farm 2, 5.00% (0.79%–0.93%) in farm 3, and 4.00% to 4.08% (0.67–0.69%) in farm 4. The mean (SD) of protein percentages were 3.08% to 3.01% (0.30%–0.31%) in farm 1; 3.33% to 3.38% (0.28%–0.29%) in farm 2; 3.68% to 3.69% (0.39%–0.40%) in farm 3; and 3.17% to 3.21% (0.31%–0.32%) in farm 4. Between the 2 breeds, Jersey cows produced higher fat and protein percentages than Holstein cows.

Fisher's and ANOVA-based ICC were computed (Table 1). For protein percentage, ICC values were generally high across the 4 farms, except for farm 2. The single-rater ICC for protein percentage was ∼0.93 in farm 1, 0.89 in farm 3, and 0.90 in farm 4, but was notably lower in farm 2 (0.58; Table 1). In contrast, the single-rater ICC for fat percentage ranged from 0.49 to 0.67 across the 3 milkings, with substantially lower values in farm 2 (0.02 to 0.10). Averaging fat and protein percentages across the 3 milkings significantly improved consistency (ICC): 0.75 to 0.86 for fat percentages and 0.96 to 0.98 for protein percentages in farms 1, 3, and 4. However, farm 2 showed lower consistency (ICC): 0.06 to 0.24 for fat percentage and 0.81 for protein percentage. Compared with the ANOVA-based ICC, Fisher's ICC aligns more closely with ICC3, where the effects of the 3 milkings are considered fixed and cow effects are random. Plots of Fisher's intraclass correlations by lactation month for fat and protein percentages across the 4 farms are presented in Figure 1.

**Table 1:** Consistency assessment of fat and protein percentages from each thrice-milking in 4 dairy farms

Type^1^	ICC (95% CI)			
	Farm 1 (Holstein)	Farm 2 (Jersey)	Farm 3 (Jersey)	Farm 4 (Holstein)
Fat percentage				
Fisher	0.606 (0.599–0.614)	0.096 (0.086–0.107)	0.495 (0.484–0.505)	0.670 (0.662–0.677)
ICC1	0.529 (0.520–0.537)	0.022 (0.012–0.032)	0.494 (0.483–0.504)	0.667 (0.659–0.674)
ICC2	0.545 (0.419–0.638)	0.080 (0.050–0.109)	0.494 (0.484–0.504)	0.667 (0.658–0.677)
ICC3	0.606 (0.598–0.614)	0.097 (0.086–0.108)	0.495 (0.485–0.505)	0.670 (0.663–0.678)
ICC1k	0.771 (0.765–0.777)	0.063 (0.035–0.090)	0.745 (0.737–0.753)	0.857 (0.853–0.861)
ICC2k	0.782 (0.684–0.841)	0.206 (0.138–0.268)	0.745 (0.737–0.753)	0.857 (0.852–0.863)
ICC3k	0.822 (0.817–0.826)	0.243 (0.221–0.266)	0.746 (0.738–0.754)	0.859 (0.855–0.863)
Protein percentage				
Fisher	0.927 (0.926–0.930)	0.583 (0.574–0.592)	0.886 (0.883–0.889)	0.901 (0.898–0.903)
ICC1	0.927 (0.925–0.929)	0.579 (0.570–0.588)	0.886 (0.883–0.889)	0.899 (0.896–0.902)
ICC2	0.927 (0.925–0.929)	0.580 (0.568–0.591)	0.886 (0.883–0.889)	0.899 (0.892–0.905)
ICC3	0.927 (0.926–0.929)	0.584 (0.575–0.592)	0.886 (0.883–0.889)	0.902 (0.899–0.905)
ICC1k	0.974 (0.974–0.975)	0.805 (0.799–0.811)	0.959 (0.958–0.960)	0.964 (0.963–0.965)
ICC2k	0.974 (0.974–0.975)	0.805 (0.798–0.813)	0.959 (0.958–0.960)	0.964 (0.961–0.966)
ICC3k	0.975 (0.974–0.975)	0.808 (0.802–0.802)	0.959 (0.958–0.960)	0.965 (0.964–0.966)

1 Fisher = Fisher's interclass correlation; ICC1–3 and ICC1–3k = ANOVA-based interclass correlations (see Equations 5 and 7–11).

**Figure 1:**
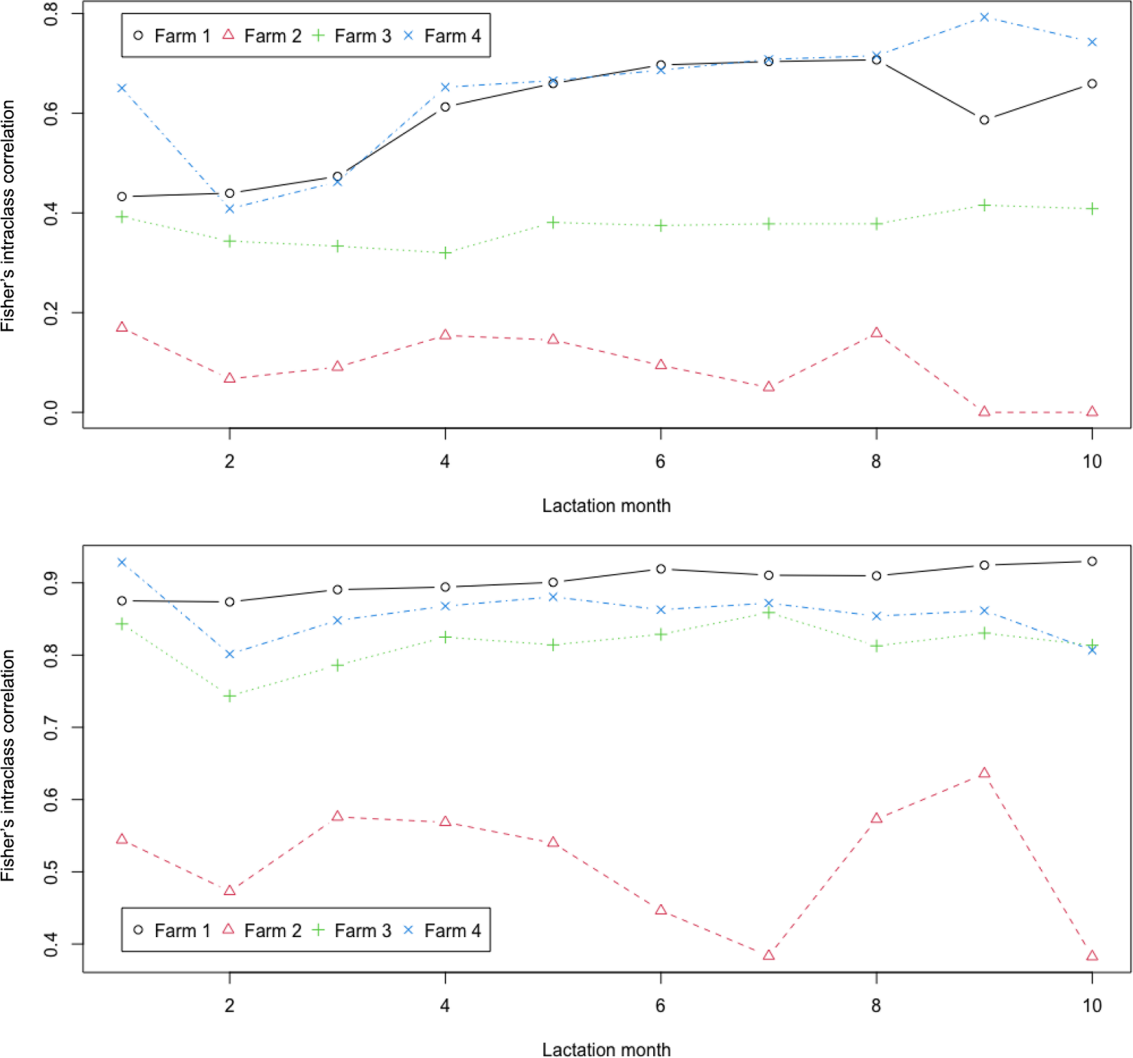
Plots of Fisher's intraclass correlations by lactation months for fat (top) and protein (bottom) percentages in 4 dairy farms (Holstein: farms 1 and 4; Jersey: farms 2 and 3).

In farm 2, pairwise simple correlations between milkings were moderate (0.375) between milkings 1 and 3, low (0.101) between milkings 2 and 3, and negative (−0.154) between milkings 1 and 2 (−0.154). In contrast, farm 3, another Jersey dairy farm, exhibited higher pairwise simple correlations: 0.458 between milkings 1 and 2, 0.431 between milkings 1 and 3, and 0.596 between milkings 2 and 3. For protein percentages, pairwise correlations were also significantly lower in farm 2 (0.563–0.584) than in farm 3 (0.871–0.914). These results align with the ICC measure, indicating potential data quality issues in farm 2 that warrant further investigations.

The interpretative guidance of ICC is practically important. According to Koo and Li (2016), ICC values are classified as poor (<0.50), moderate (0.50–0.75), good (0.75–0.90), and excellent (>0.90). A more lenient classification by Cicchetti (1994) defines ICC values as poor (<0.40), fair (0.40–0.59), good (0.60–0.74), and excellent (0.75–1.00). Consequently, ICC values below 0.4 or 0.5 often indicate poor data quality. However, applying a universal threshold to biological traits such as fat and protein percentages may be debatable, as their genetic determinations differ. Instead, trait-specific minimum ICC thresholds (e.g., established through bootstrapping) are preferred. To illustrate, we combined the milking data from the 2 Holstein farms and computed the 95% CI via 10,000 times of bootstrapping. Each bootstrap sample (replicate) was generated by sampling with replacement, retaining the same number of milking records for an average herd size (317) based on unique cow IDs. This sample size corresponds to the average herd size of dairy cows (337) in the United States in 2022 (O'Leary, 2023). The 95% CI for Fisher's ICC were 0.584 to 0.762 for fat percentages and 0.900 to 0.952 for protein percentages. Thus, a plausible threshold for “good” consistency can be set at ∼0.58 for fat percentages and 0.90 for protein percentages for Holstein cattle. It should be noted that this is only an illustration. More precise thresholds for each dairy breed can be determined through a comprehensive data analysis incorporating random samples across multiple geographic regions, herds, years, and lactations.

Furthermore, daily fat and protein percentages were calculated as weighted averages of the percentages from each milking, with weights being the proportional partial daily yields for the 3 milkings per cow. To assess accuracy, we examined the intercept and slope of the linear regression of daily fat and protein percentages (*y*) against the percentages from each milking (*x*) (Table 2). For an ideal accuracy assessment, *a* should be close to zero, indicating no systematic bias in the predictions. Across the 4 daily farms, intercept values for fat percentages deviated substantially from 0, ranging from 0.94 to 2.24. This finding suggests potential systematic biases if partial-yield fat percentages are used as proxies for daily yield fat percentages, indicating the need for adjustment across multiple milkings daily. In contrast, intercept values for protein percentages were close to zero (0.13–0.29), except in farm 2 (0.68–0.89).

**Table 2:** Accuracy assessment for using single-milking fat and protein percentages as proxies of daily yield fat and protein percentages in 4 dairy farms^1^, ^2^

Farm	Fat percentage				Protein percentage			
	*a*	*b*	MSE	R^2^	*a*	*b*	MSE	R^2^
1								
Milking 1	1.208 (0.014)	0.730 (0.004)	0.162	0.541	0.133 (0.006)	0.959 (0.002)	0.005	0.943
Milking 2	1.118 (0.014)	0.740 (0.003)	0.131	0.627	0.144 (0.005)	0.953 (0.002)	0.004	0.954
Milking 3	0.940 (0.015)	0.718 (0.003)	0.209	0.407	0.175 (0.005)	0.941 (0.002)	0.005	0.948
2								
Milking 1	2.241 (0.016)	0.289 (0.003)	0.727	0.019	0.695 (0013)	0.601 (0.004)	0.024	0.590
Milking 2	2.358 (0.017)	0.310 (0.004)	0.539	0.015	0.682 (0.014)	0.596 (0.004)	0.025	0.574
Milking 3	2.286 (0.020)	0.314 (0.004)	0.230	0.082	0.886 (0.015)	0.539 (0.004)	0.021	0.647
3								
Milking 1	1.924 (0.024)	0.620 (0.005)	0.270	0.362	0.275 (0.010)	0.926 (0.003)	0.015	0.894
Milking 2	1.540 (0.021)	0.690 (0.004)	0.185	0.563	0.275 (0.008)	0.925 (0.002)	0.011	0.927
Milking 3	1.611 (0.021)	0.674 (0.004)	0.202	0.524	0.278 (0.008)	0.925 (0.002)	0.010	0.930
4								
Milking 1	0.927 (0.015)	0.770 (0.004)	0.103	0.712	0.167 (0.007)	0.953 (0.002)	0.006	0.935
Milking 2	0.925 (0.016)	0.767 (0.004)	0.117	0.673	0.285 (0.008)	0.905 (0.002)	0.010	0.894
Milking 3	0.923 (0.014)	0.784 (0.003)	0.098	0.724	0.176 (0.006)	0.945 (0.002)	0.005	0.949

1 *a*, *b* = intercept and regression coefficient; $R^{2}=\frac{Var\left(y\right)-MSE}{Var\left(y\right)},$ where *Var* (*y*) represents the phenotypic variance of percentage daily milk components, and *MSE* is mean squared error.

2 For *a* and *b*, values are presented as the estimate, with SE in parentheses.

The regression slope (*b*) represents the change in the actual value (*y*) for a unit change in the predicted value (*x*). Ideally, if *x* perfectly predicts *y*, *b* should equal 1. Across the 4 daily farms, the regression slopes for protein percentage were close to 1 (0.91–0.96), except in farm 2 (0.54–0.60), suggesting minimal systematic biases if using partial-yield protein percentages as proxies of daily yield protein percentages. The mean squared errors (**MSE**) for projected daily yield protein percentages were very low (0.004–0.015) in farms 1, 3, and 4, but higher (0.021–0.025) in farm 2, likely due to data quality issues. In contrast, the regression slope for fat percentage was substantially below 1, ranging from 0.73 to 0.78 in farms 1, 3, and 4, and from 0.29 to 0.31 in farm 2. A low regression slope for fat percentage led to under-predicted daily yield fat percentages. As a result, MSE values for fat percentages were notably larger (0.94–2.29) across the 4 dairy farms compared with protein percentages.

An F-test revealed that the variance of observed milk fat and protein percentages in farm 2 was significantly lower than in farm 3, another Jersey farm (*P* < 2.2 × 10^−16^). The 95% CI of the variance ratios between the 2 farms were 0.572 to 0.614 for milk fat percentage and 0.392 to 0.421 for milk protein percentage. In contrast, milk fat and protein variances did not differ significantly between the 2 Holstein farms (*P* = 0.322 and *P* = 0.164, respectively), with the 95% CI of 0.952 to 1.02 (milk fat) and 0.946 to 1.01 (milk protein) for the variance ratio between the 2 farms. Concerning the potential cause for the data problem in farm 2, sample ID mismatches during fat and protein assessments are possible but unlikely to substantially reduce variance. Instead, systemic errors such as inadequate milk mixing before sampling or calibration errors in measurement equipment may be more plausible contributors. For example, if the equipment for measuring fat and protein percentages was improperly calibrated or faulty, it could systematically report a narrower range of values, reducing variances. Although additive and independent measurement errors typically increase variance, systematic or multiplicative errors, particularly those with adverse scaling effects, can reduce variance. Additionally, clipping due to device range limits could artificially constrain values, particularly for fat percentages, which are inherently more variable.

Generalized additive models were employed to examine the effects of key variables on fat and protein percentages. The results revealed similar patterns for fat and protein percentages across the 4 farms. For instance, the results obtained from farm 4 are illustrated in the graphical abstract. The effects of lactation number exhibited roughly a quadratic polynomial pattern, peaking in the second lactation. The effects of milking interval time remained relatively stable between 7 and 9 h but showed drastic variations beyond this range. The impact of days in milking declined sharply in the first 2 mo of lactation and increased gradually afterward until the end of lactation. Finally, milking numbers showed significant effects on fat and protein percentage, which were primarily related to varied milk yields across the 3 milkings, given the 3 relevant variables included in the model already. Overall, all these effects are significant, showing nonlinear patterns. Hence, adjustments accounting for the difference in milking intervals or days in milk alone may not be sufficient, and more accurate adjustments may require considering nonlinear calibrations accounting for all key affecting variables (Liu et al., 2020; Gerke et al., 2025).

In conclusion, we evaluated the consistency of single-milking fat and protein percentages across thrice-daily milkings. Our results indicate high consistency of protein percentages but potentially relatively lower consistency with fat percentages across thrice-daily milkings. Therefore, applying varied adjustments for fat percentages across multiple milkings is plausible, and nonlinear adjustments may be more accurate than linear calibrations while accounting for the effects of key variables such as milk interval time, DIM, and parity. Adopting alternative sampling or estimating fat and protein percentage from the mixed daily milk are also plausible solutions. To assess the consistency of intraday fat and protein percentages, pairwise correlations analyze relationships between 2 measurements at a time, leading to multiple separate correlation coefficients that do not provide a single summary statistic for overall agreement. In contrast, ICC aggregates information across all repeated measures, providing a single reliability estimate that reflects overall consistency.
